# ACUM, an easily underdiagnosed cause of dysmenorrhea—A case report

**DOI:** 10.3389/fmed.2024.1308299

**Published:** 2024-01-26

**Authors:** Qicai Hu, Chunlei Guo, Qing Chen, Wei Zhang, Huifang Wang, Weixia Wei

**Affiliations:** ^1^Center of Obstetrics and Gynecology, Peking University Shenzhen Hospital, Shenzhen, China; ^2^Shenzhen Peking University-Hong Kong University of Science and Technology (PKU-HKUST) Medical Center, Institute of Obstetrics and Gynecology, Shenzhen, China; ^3^Shenzhen Key Laboratory on Technology for Early Diagnosis of Major Gynecologic Diseases, Shenzhen, China

**Keywords:** accessory cavitated uterine malformation, dysmenorrhea, ultrasound, laparoscopy, surgery

## Abstract

This report described three cases with long-term severe dysmenorrhea, and these cases were referred to our department for medical help. The diagnosis of accessory cavitated uterine malformation (ACUM) was considered based on symptoms and ultrasound/MRI findings. Moreover, a combined approach involving hysteroscopic surgery and laparoscopic surgery was undertaken, and no complications occurred during surgery and in the post-operative period. It is important to consider ACUM in patients with severe dysmenorrhea that does not respond to medical management. Surgery is the most effective treatment for this anomaly.

## Introduction

Accessory cavitated uterine malformation (ACUM) is characterized as a cystic uterine lesion lined with functional endometrium and surrounded by a thick layer of the myometrium. ACUM is usually formed at the lateral aspect of the myometrium, just beneath the insertion of the round ligament. ACUM was mainly diagnosed in women with the age of below 30 years and rarely reported in multipara women. The symptoms of ACUM may manifest as early as at the onset of menarche or shortly after that (1). Dysmenorrhea unresponsive to medical therapy, along with pelvic pain and dyspareunia, represents the primary symptoms. Pain often tends to increase following the onset of menses, though it may present asymptomatically in certain women. Either anomaly or deformity of a novel Müllerian tube related to a gubernaculum dysfunction has been considered as a pathology of ACUM (2). The clinical understanding of these lesions is currently limited, with no published population-based studies. The prevalence of ACUM remains unknown due to its rarity, potentially leading to significant underestimation and underdiagnosis. ACUM is often misdiagnosed as degenerated leiomyoma, cystic adenomyoma, or uterine malformations such as bicornuate uterine (3). In this report, we examined three newly diagnosed ACUM cases in 2023 to enrich the knowledge of ACUM for gynecologists and to facilitate a better management of this anomaly.

## Informed consent

This study protocol was approved by the institutional review board (IRB) of Peking University Shenzhen Hospital and in accordance with the ethical standards formulated in the Helsinki Declaration. Informed consent was obtained from all participants before enrollment.

### Case 1

A 30-year-old woman had experienced severe progressive dysmenorrhea for the past 13 years. The woman utilized a 10-point visual analog scale (VAS) to self-report the severity of her dysmenorrhea and non-steroidal anti-inflammatory drugs (NSAIDs) during her mensural period. Over the past 9 years, the woman underwent several ultrasound examinations and the diagnosis of uterine fibroid was considered. However, no specific treatment was undertaken. Uterine fibroid or adenomyoma was diagnosed based on the ultrasound findings performed in our hospital 3 years ago. Subsequently, the patient was advised to consider getting pregnant as soon as possible. The pregnancy was delivered by cesarean section, and during the surgery, a mass was discovered on the uterine wall. However, no resection of the mass was performed at that time. Following resumption of menses, dysmenorrhea recurred, necessitating treatment.

Ultrasound performed in our hospital at this time revealed a well-circumscribed heterogeneous anechoic area located at the right lateral wall of the myometrium, beneath the uterine horn. An annular vascular thick rim surrounded this area. Hypoechoic ground-glass opacities were observed within the area. On MRI, a mass with a measurement of 28 × 25 × 31 mm was identified at the right lateral aspect of the uterus. The T1-weighted sequence showed a short signal, while the T2-weighted sequence exhibited a long signal, both concentrated in the center of the mass. The surrounding signal and an enhancement scan signal corresponded to the myometrium. The structure of endometrium, uterine junctional zone, and myometrium was clearly visible.

**Figure 1:**
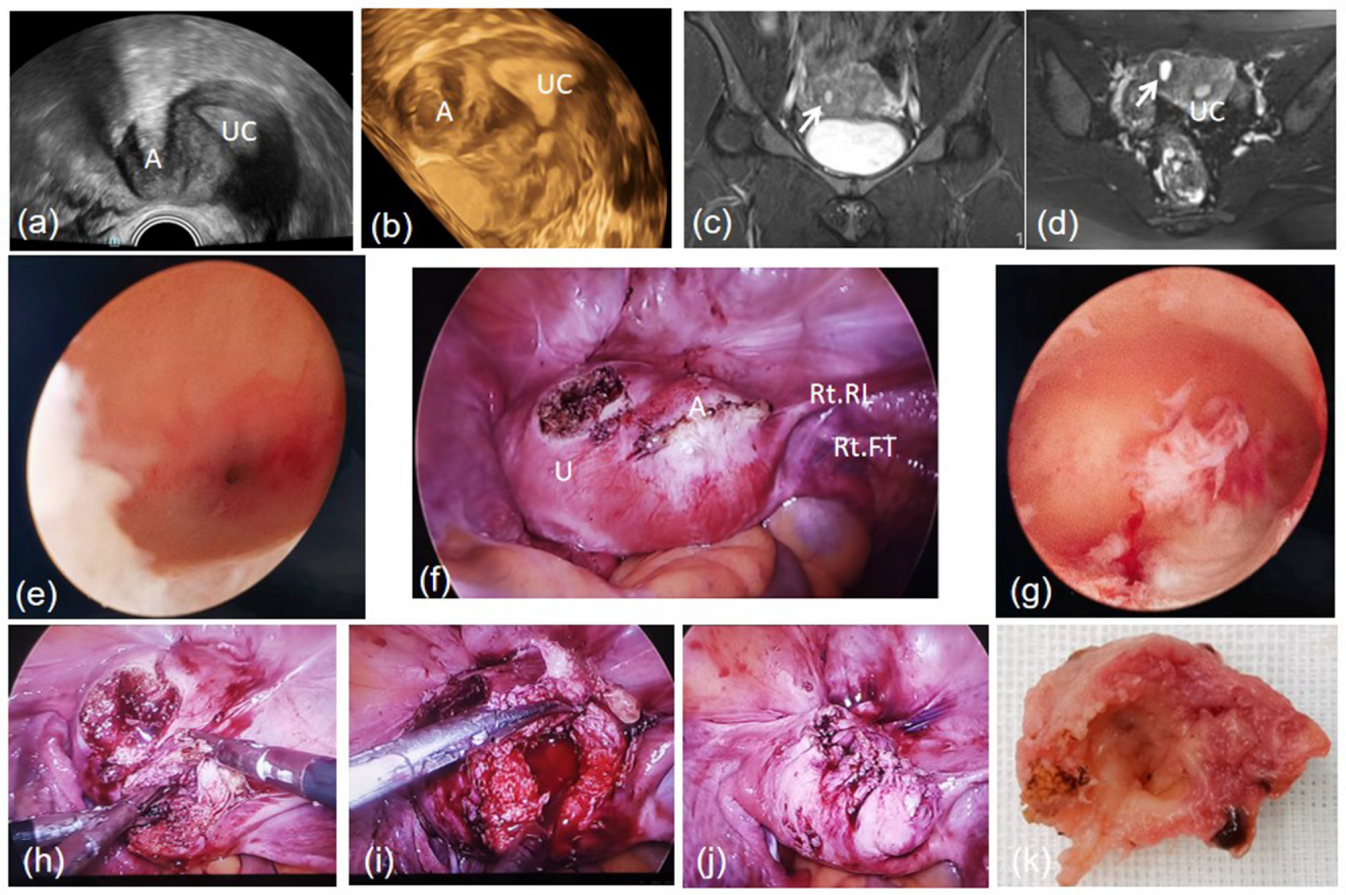
A cavity mass beneath the right uterine horn in transvaginal ultrasound **(a, b)**. MRI T2-weighted sequence in a coronal view **(c)** and an axial view **(d)**. Left oviductal orifice **(e)**. ACUM located beneath the right round ligament in the laparoscopic view **(f)**. Normal uterine cavity **(g)**. Blunt dissection of the cavity mass **(h)**. The uterine after cavity mass removed **(i)**. Suture the myometrium **(j)**. The cystic wall **(k)**. A, Accessory cavitated uterine malformation; U, uterus; UC, uterine cavity; Rt RL, right round ligament; Rt FT, right fallopian tube.

A combined hysteroscopic and laparoscopic surgery was performed. No abnormalities were found in the diagnostic hysteroscopic surgery. However, in the laparoscopic surgery, uterus was found asymmetrically enlarged and a mass was found in myometrium beneath the right round ligament. Both side uterine horns, fallopian tubes, and ovaries appeared normal in visual inspection. Firstly, a combination of 6 u pituitrin and 20 u oxytocin (diluted in 10 ml saline) was administered at the boundary of the mass and myometrium to minimize the amount of bleeding during and after the surgery. Following the transverse incision over the surface by the monopolar coagulation hook, a colored fluid was observed to emerge. Then, the mass was completely resected. Notably, the boundary was not as clear as that typically observed between leiomyoma and myometrium. In the end, 1-0 absorbable barb was used to suture the myometrium. The pathological examination confirmed the diagnosis of ACUM. No complications occurred during peri-operative period, and the patient was discharged 2 days after surgery. According to the 3-month follow-up record, no dysmenorrhea was reported after surgery (Figure 1).

### Case 2

A 25-year-old non-pregnant woman with remarkable face acne was referred to our department due to long standing dysmenorrhea. The patient experienced menarche at the age of 13 years and had been dealing with dysmenorrhea since that time. The mensural period was irregular, spanning from 30 to 90 days. Dysmenorrhea, resistant to NASIDs, lasted as long as 2 weeks after menstruation for the past 8 years. The self-reported VAS for pain severity reached up to 10 points. Ultrasound examinations performed in several other hospitals revealed suspected uterine fibroid or adenomyoma while no additional treatment was undertaken due to uncertainty diagnosis.

Ultrasound findings showed polycystic ovaries along with a well-circumscribed heterogeneous anechoic area surrounded by a thick rim. This area was located at the right lateral aspect of the myometrium, just beneath the uterine horn. During pelvic MRI, a quasi-circular cavity mass measuring 22 × 27 × 30 mm was observed at the right lateral aspect of the uterine in myometrium. The mass exhibited an isointensity signal in T1-, T2-, and DWI-weighted images, while presenting a hypointensity signal in ADC-weighted images. The enhancement scan signal corresponded to the myometrium, while no reinforcement signal was shown in the cavity.

**Figure 2:**
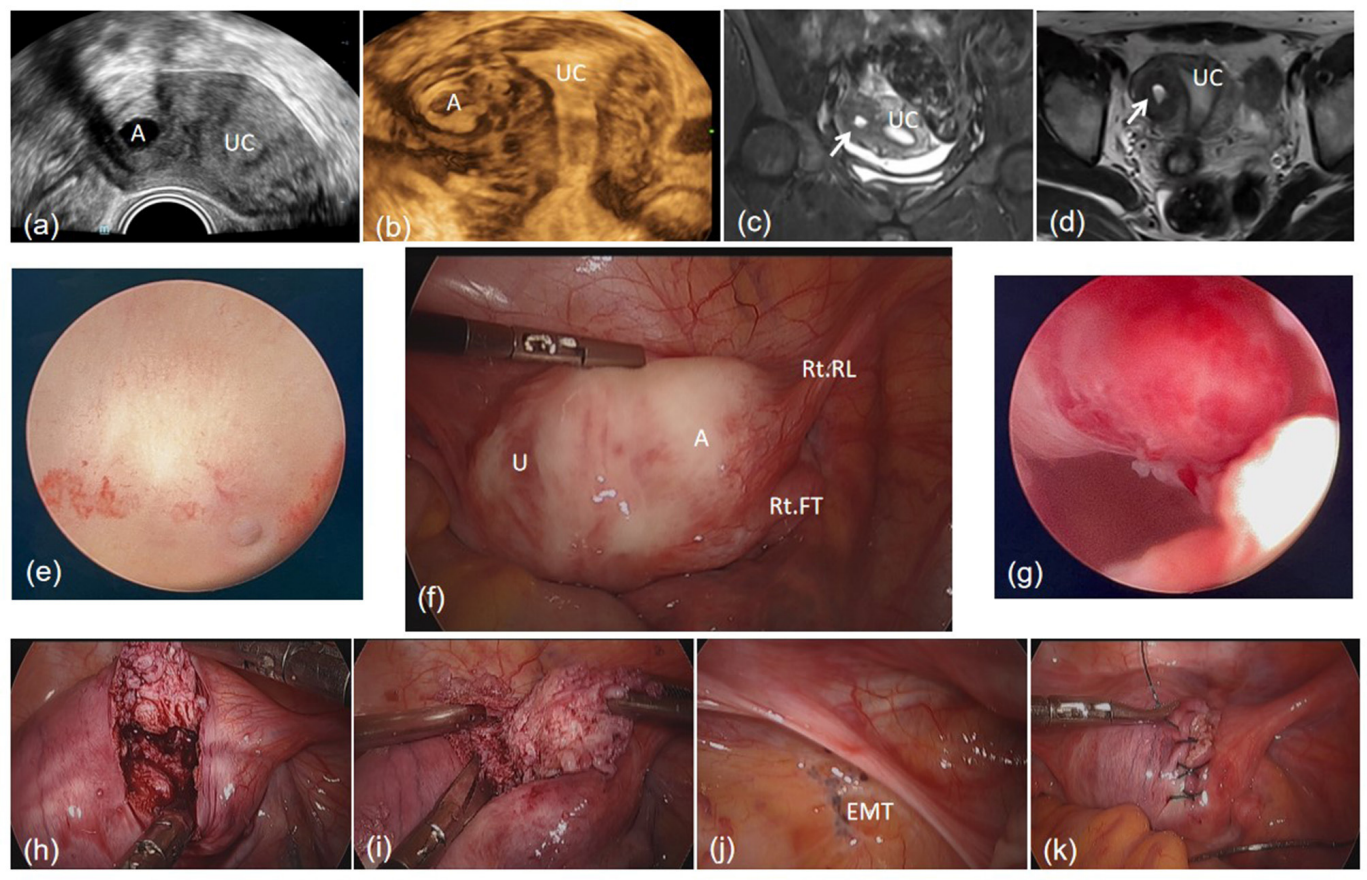
A cavity mass at right of the uterine in transvaginal ultrasound **(a)**. The mass in 3-D modality of transvaginal ultrasound **(b)**. MRI T2-weighted sequence in a coronal view **(c)** and an axial view **(d)**. Left oviductal orifice **(e)**. The mass beneath the right round ligament in the laparoscopic view **(f)**. A small polypoid mass in uterine cavity **(g)**. Blunt dissection of the mass **(h, i)**. Endometriosis lesions in the peritoneum of the right pelvic wall **(j)**. Suture of the myometrium **(k)**. A, Accessory cavitated uterine malformation; U, uterus; UC, uterine cavity; Rt RL, right round ligament; Rt FT, right fallopian tube; EMT, endometriosis.

Except a small polypoidal mass, no other abnormalities were identified in the diagnostic hysteroscopic surgery. However, in the laparoscopic surgery, a spherical enlargement was found at the lateral aspect of the myometrium, beneath the right uterine horn; furthermore, endometriotic lesions were observed over the peritoneum of the right pelvic wall. Pituitrin and oxytocin were injected; subsequently, a longitudinal incision was made on the surface of the sphere using the monopolar coagulation hook; and the lesions and myometrium were clearly demarcated. During the removal, chocolate-colored fluid discharged from sphere; nonetheless, a radical resection was performed. Finally, 1-0 absorbable barb was used to suture the myometrium. The cystic wall was well-differentiated smooth muscle lined with endometrial glands and stroma according to the pathological examination, confirming the diagnosis of ACUM. No complications occurred in the peri-operative period, and the patient was discharged 3 days after surgery. According to the 3-month follow-up record, no dysmenorrhea was reported after menstruation resumed (Figure 2).

### Case 3

A 30-year-old non-pregnant woman reported to our department seeking medical advice for serious hypogastralgia during the peri-ovulation period. The increase in severity of dysmenorrhea started 5 years ago with sensation of rectal tenesmus. The VAS was self-reported up to 10 points, and the pain was also unresponsive to the painkiller. An ultrasound examination performed 3 years ago reported suspected uterine myoma while previous examination reported no abnormality. No surgery or any other aggressive management was taken ever before.

An ultrasound performed in our hospital revealed a well-circumscribed heterogeneous anechoic area surrounded by a thick rim in the myometrium. This area was located at the lower-left anterior wall. MRI revealed a round lesion with a measurement of 34 × 33 × 28 mm in the lower part of the left uterine wall with an isointensity signal in both T1-weighted and T2-weighted images, similar to the signal of myometrium. Additionally, the enhancement scan signal demonstrated the similar characteristics.

**Figure 3:**
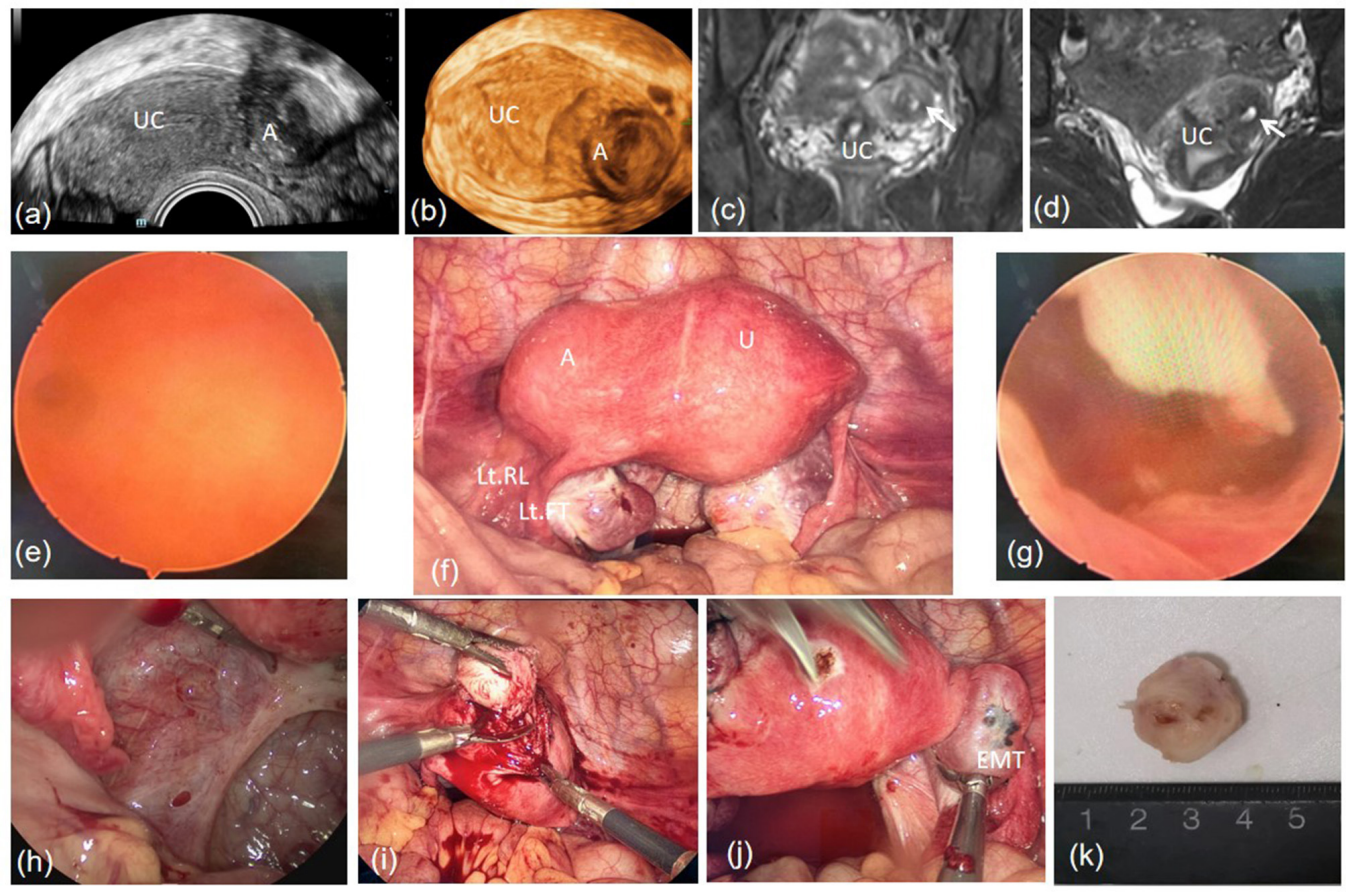
A well-circumscribed heterogeneous anechoic area surrounded by a thick rim in the left lateral aspect of the myometrium **(a, b)**. A cavity mass at the left bottom of uterine with an isointensity signal in T1-weighted and T2-weighted, corresponded to the signal of myometrium **(c, d)**. Right oviductal orifice **(e)**. The mass was located beneath the left round ligament in the laparoscopic view **(f)**. Normal uterine cavity **(g)**. A window-liked structure in the peritoneum of the left pelvic wall in the laparoscopic view **(h)**. Radical resection **(i)**. Endometriosis lesions on the right ovary **(j)**. The section of the mass was cavity-liked **(k)**. A, Accessory cavitated uterine malformation; U, uterus; UC, uterine cavity; Lt RL, left round ligament; Lt FT, left fallopian tube; EMT, endometriosis.

No abnormalities were found in diagnostic hysteroscopic surgery. However, in laparoscopic surgery, the lesion was appeared at the left lateral aspect of the myometrium, inner side of the round ligament. Several purple-blue endometriotic lesions on the right ovary and a window-liked structure in the peritoneum of the left pelvic wall were also observed. A longitudinal incision was made on the surface of the lesion using the monopolar coagulation hook following the injection of pituitrin and oxytocin. The lesions and myometrium were clearly demarcated and led to a radical resection. The excised mass was round and gray with a thick wall, and the cavity was filled with chocolate-colored fluid. The pathological examination confirmed the diagnosis of ACUM, revealing the cavity lined with functional endometrium glands and stroma, as well as in myometrium. The patient was discharged 3 days after surgery. According to the 3-month follow-up record, no dysmenorrhea was reported after resumption of menses (Figure 3).

## Discussion

ACUM was called accessory cavitated uterine mass or juvenile cystic adenomyomas previously, described as isolated cavitated lesions in the myometrium, non-communicating with uterine cavity, commonly observed in women under 30 years of age and nullipara, and usually measured ≥10 mm. Cavity is filled with menstrual fluid and blood. The cyst wall was a uterine smooth muscle and lined with functional endometrium glands and stroma. Recent study demonstrated that ACUM can be found in adult women (1). Therefore, the word “juvenile” used in the terminology might cause confusion. Despite both ACUM and juvenile cystic adenomyomas being myometrial pathologies, they share the characteristic of having endometrial glands and stroma within the myometrium. Moreover, adenomyosis have highly variable morphological appearance while ACUM have very consistent appearance and lined with endometrial glands and stroma in the cyst (4). Using the word “malformation” rather than “mass” can better explain the pathogenesis of the lesion. In recent years, the hypothesis of congenital developmental anomaly of the Müllerian tube was accepted by many researchers. In 2021, the terminology of “accessory cavitated uterine malformation” was recommended as the description of the cystic lesions in the myometrium with specific location, typical symptoms, and characteristic morphological imaging features (1). The widely accepted diagnostic criteria were, proposed by Acién et al., as follows (2): (1) isolated accessory cavitated mass; (2) normal uterus (with a cavity lined by an functional endometrium), fallopian tubes and ovaries; (3) pathological confirmation after surgical excision; (4) the cavity filled with chocolate-colored fluid; (5) without a true adenomyosis; (6) accessory cavity lined by functional endometrium with glands and stroma; and (7) concentric arrangement of smooth muscle in myometrial mantle.

Severe dysmenorrhea, which is non-responding to painkiller, and recurrent pelvic pain are the major symptoms of ACUM. However, the symptoms are common and non-specific in women of childbearing age, often leading to ignorance by both patients themselves and gynecologists. Furthermore, the majority of lesions of ACUM commonly measured ≤ 50 mm, according to certain reports (1, 5). The mean external diameter was 22.8 mm (95%CI 20.9–24.8 mm), and the mean internal diameter was 14.1 mm (95%CI 12.2–16.1 mm). The size small of lesion can make it difficult to distinguish from uterine fibroid, fibroid cystic change, or adenomyosis in ultrasound examinations. As shown in our three cases, ACUM was also wrongly diagnosed as uterine fibroid degeneration and adenomyosis. So far, 5 cases were reported with an interval of >8 years between symptom onset and diagnosis and the longest gap was 24 years (3, 5–8). In our study, the gap between the onset of dysmenorrhea and surgery of the three cases was 13, 12, and 5 years, respectively. However, the gap between the abnormalities found in ultrasound and surgery was 9, 8, and 3 years, respectively. The extended time gap was believed to be related to insufficient knowledge and poor understanding of ACUM.

Case 2 and case 3 were concerned for diagnosis with pelvic endometriosis (for stage I, the ASRM score was 2 and 5 points, respectively), another common cause of dysmenorrhea in women of childbearing age. Endometriotic lesions can be found in patients diagnosed with ACUM on various locations, including the uterosacral ligaments, the posterior peritoneum of broad ligaments, rectum, uterine surface, and even in ovaries (7, 9), indicating that ACUM should kept in mind as a diagnosis in childbearing age women with severe dysmenorrhea, even if diagnosis of endometriosis is considered.

The characteristics of ACUM had been documented with the understanding of the anomaly and the development of the ultrasound examination (5). Transvaginal ultrasound is regarded as the first-line choice for ACUM diagnosis in consideration of its convenience, accuracy, non-invasive nature, and cost-benefit. Utilizing full capabilities of 3-D transvaginal ultrasound, an intra-myometrial rounded cystic image with sharp edges and finely echogenic fluid content can be clearly visualized. Transrectal ultrasound may be considered, especially for nulliparous. MRI provides an effective method for ACUM diagnosis, showing an intra-myometrial hemorrhagic cystic image and a precise relationship with myometrium and/or endometrium without any contrast injection (9). A hypersignal on T1- and T2-weighted sequences reflects a hemorrhagic content, while hypointensity of the surrounded tissue on T2-weighted is the typical aspect of the fibrous tissue.

While certain cases report the diagnosis of ACUM in infertility women (7), there is no definitive evidence indicating a direct association between this anomaly and reproductive health issues. The published study of ACUM mainly focused on case series, lack of population-based longitudinal observational study, and no reoccurred report after surgery. The natural history of ACUM still remains imprecise.

In accordance with the severity of the symptoms, the management of ACUM included expectant management, NSAIDs, oral contraceptive pills (OCP), levonorgestrel intrauterine system (LNG-IUS), GnRH agonist therapy, mass excision, and hysterectomy (1, 3, 10). Dysmenorrhea can be relived to the certain extent following a 12-month course of hormonal therapy; however, the relief is limited and dysmenorrhea may occur shorty after withdrawal of hormonal treatment. Hence, this therapy is not recommended for the young women with severe dysmenorrhea. Instead, surgery is considered as the most effective treatment for ACUM. Fertility preservation should be taken into consideration before surgical management, and laparoscopic minimally invasive surgery is the preferable treatment option (11). The lesions of ACUM commonly located at the lateral aspect of the myometrium, beneath the insertion of the round ligament, leading to recommendation of anterior wall uterine incision rather than incision on posterior wall, thereby reducing the possibility of injury to ascending branch of the uterine artery. As the boundary between the lesions and myometrium is usually clearly demarcated, the resection is similar to laparoscopic myomectomy. Urinary tract anomalies must be ruled out before surgery as they are associated with mullerian anomalies to reduce the chance in urological injuries (2).

## Conclusion

ACUM is one of the causes of dysmenorrhea in young women. This anomaly may be underestimated and underdiagnosed due to rare presentation, non-specific symptoms, and small volume of the mass. ACUM should be considered patients with severe dysmenorrhea resistant to medical management. Early diagnosis and appropriate management via laparoscopic minimally invasive surgery can significantly improve the patient's quality of life.

## Data availability statement

The original contributions presented in the study are included in the article/Supplementary material, further inquiries can be directed to the corresponding author.

## Ethics statement

The studies involving humans were approved by Institutional Review Board (IRB) of Peking University Shenzhen Hospital. The studies were conducted in accordance with the local legislation and institutional requirements. The participants provided their written informed consent to participate in this study. Written informed consent was obtained from the individual(s) for the publication of any potentially identifiable images or data included in this article.

## Author contributions

QH: Writing – original draft, Writing – review & editing. CG: Writing – original draft, Writing – review & editing. QC: Writing – review & editing. WZ: Writing – review & editing. HW: Writing – review & editing. WW: Writing – original draft, Writing – review & editing.
