# Supplementary material for: ACUM, an easily underdiagnosed cause of dysmenorrhea—A case report
Source: Front Med (Lausanne). 2024 Jan 26;11:1308299. doi: 10.3389/fmed.2024.1308299 (PMC10853427; doi:10.3389/fmed.2024.1308299)
Supplement: Supplementary file 1 [file Table_1.DOCX]

Table 1 Characteristics of the 3 cases

| No. of cases | Age of menarche  (year) | Age at the onset of symptoms  (year) | Age at abnormal imaging (year) | Age at operation  (year) | gravidity | parity | Lesion size(mm)-outer capsule(measured by MRI ) | Lesion site | Previous diagnosis | Treatment |
| --- | --- | --- | --- | --- | --- | --- | --- | --- | --- | --- |
| 1 | 14 | 17 | 21 | 30 | 1 | 0 | 31 | R | Leiomyoma  Degenerated leiomyoma  adenomyoma | Laparoscopic Resection of ACUM |
| 2 | 13 | 13 | 17 | 25 | 0 | 0 | 30 | R | leiomyoma  adenomyoma | Laparoscopic Resection of ACUM |
| 3 | 13 | 25 | 25 | 30 | 0 | 0 | 34 | L | leiomyoma | Laparoscopic Resection of ACUM |
